# Fast reconstruction of 3D volumes from 2D CT projection data with GPUs

**DOI:** 10.1186/1756-0500-7-582

**Published:** 2014-08-30

**Authors:** Miriam Leeser, Saoni Mukherjee, James Brock

**Affiliations:** Department of Electrical and Computer Engineering, 440 Dana Building, Northeastern University, 360 Huntington Ave, Boston, MA 02115 USA; Cognitive Electronics, 201 South St, Suite 301, Boston, MA 02111 USA

**Keywords:** Computed tomography, Graphics processing unit, Conebeam reconstruction, CUDA, OpenCL

## Abstract

**Background:**

Biomedical image reconstruction applications require producing high fidelity images in or close to real-time. We have implemented reconstruction of three dimensional conebeam computed tomography(CBCT) with two dimensional projections. The algorithm takes slices of the target, weights and filters them to backproject the data, then creates the final 3D volume. We have implemented the algorithm using several hardware and software approaches and taken advantage of different types of parallelism in modern processors. The two hardware platforms used are a Central Processing Unit (CPU) and a heterogeneous system with a combination of CPU and GPU. On the CPU we implement serial MATLAB, parallel MATLAB, C and parallel C with OpenMP extensions. These codes are compared against the heterogeneous versions written in CUDA-C and OpenCL.

**Findings:**

Our results show that GPUs are particularly well suited to accelerating CBCT. Relative performance was evaluated on a mathematical phantom as well as on mouse data. Speedups of up to 200x are observed by using an AMD GPU compared to a parallel version in C with OpenMP constructs.

**Conclusions:**

In this paper, we have implemented the Feldkamp-Davis-Kress algorithm, compatible with Fessler’s image reconstruction toolbox and tested it on different hardware platforms including CPU and a combination of CPU and GPU. Both NVIDIA and AMD GPUs have been used for performance evaluation. GPUs provide significant speedup over the parallel CPU version.

## Findings

### Introduction

CT imaging is one of the most used diagnostic methods in interventional and minimally invasive surgeries [1]. As the importance of the access to medical imagery before or during surgical procedures increases, the computational need for CT imaging becomes more demanding and challenging. It requires producing high fidelity images in or close to real-time to avoid interruptions during the treatment of patients. Conebeam CT is used to acquire knowledge of parts of the human body to obtain a clear image during/before performing a procedure. Today, most conebeam CT scanners use the Feldkamp Davis Kress algorithm [2] as the standard reconstruction method. The method takes a slice of the target, weights the projection data and then filters the weighted data before backprojecting and creating the final three dimensional image. The last step, backprojection, is the most computationally intensive with a complexity of *O*(*N*^4^) in the spatial domain and it is the bottleneck [3]. Researchers have used different architectures to accelerate this process including Application Specific Integrated Circuits (ASICs) and Field Programmable Gate Arrays (FPGAs). However, the expensive nature of these boards along with the steep learning curve necessary to program these devices often limit their use. Graphics Processing Units (GPUs) offer an alternative approach for accelerating computationally intensive jobs. Algorithms such as CT image reconstruction with intensive computation and massive data parallelism are particularly well suited for GPUs.

A popular image reconstruction toolbox, provided by Fessler [4], consists of a collection of open source algorithms for image reconstruction written in MATLAB. We have implemented the FDK algorithm from this toolbox using several different methods including single threaded code written in C, parallel code written in C with OpenMP constructs, parallel code in MATLAB using the parallel computing toolbox (PCT) and GPU codes written in CUDA-C and OpenCL. The purpose of this study is to explore the performance of these implementations on different architectures. These codes are run on two types of architectures including CPU and a combination of CPU and GPU. We have tested our implementations on both NVIDIA and AMD GPUs using both a mathematical phantom and mouse scan data.

The main contributions of this paper are:

- Our implementations are compatible with Fessler’s image reconstruction toolbox [4], a popular toolbox of open source algorithms for reconstruction of images written in MATLAB. We use the same input files and same general approach as Fessler in our implementations.
- Our implementations are tested on two types of hardware platforms: CPU and a combination of CPU and GPU. The performance has been evaluated using GPUs from two different vendors: NVIDIA and AMD.
- The performances of two complete GPU implementations of the same approach are compared, in CUDA-C and OpenCL, to serial and multithreaded C and MATLAB implementations.
- Our NVIDIA CUDA code is compatible with NVIDIA’s CUDA compiler, while other open source software is not. Our OpenCL implementation is optimized and complete.

Our code is available open source [5].

### Background

This section describes the FDK method along with a brief introduction to GPU computing and recent advances in the GPU computing model. We also discuss related work that aims at accelerating FDK using GPU, CPU, or other heterogeneous architectures.

#### FDK method

The FDK method, published by Feldkamp, Davis and Kramp in 1984 [2], introduced a method to reconstruct a 3D volume from multiple 2D projections. Here a scanner along with a 2D detector takes a full rotation around the patient or object of interest to capture the data. In this process, called conebeam scanning, the trajectory of the source is circular and each horizontal row of detector values is ramp filtered and considered as a two dimensional object. These filtered projections are then backprojected along the original rays. During the process of acquiring scanned data, the X-ray source moves in a circular orbital path, which has a radius *r*. The detector plane stands perpendicular to the rotational axis of the source and moves with it. It produces a set of projections *P*_1_,*P*_2_,…,*P*_*K*_ at *K* discrete positions of the source with uniform angular spacing. Sometimes there are mechanical limitations that preclude a full rotation from being completed.

**Figure 1:**
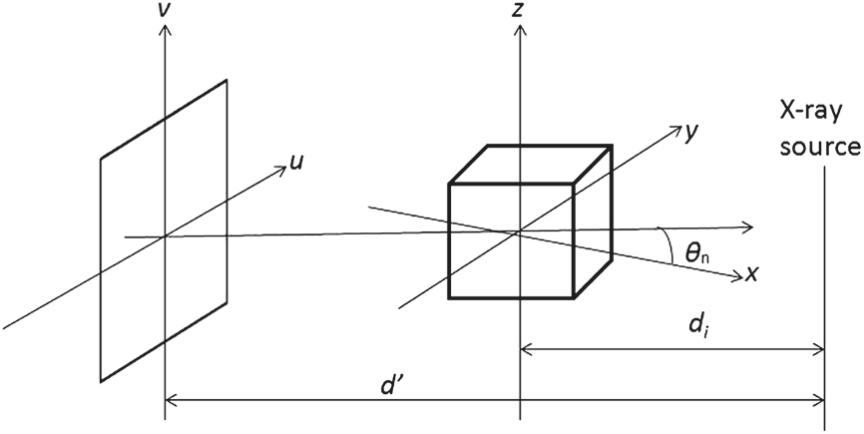
**Co-ordinate system for backprojection.**

The method can be conceptualized as a reconstruction with weighted backprojection. It is performed in two stages. First, the raw data is individually weighted and ramp filtered to produce filtered projections *Q*_1_,*Q*_2_,…,*Q*_*K*_. These projections are collected at a distance from X-ray source to detector *d*^′^ with angle *θ*_*n*_ where 1≤*n*≤*K*. The distance between the volume origin and the source is denoted *d*_*i*_. Let *F*_*x*,*y*,*z*_ represent the value of voxel (*x*,*y*,*z*) in volume *F* (Figure 1). The volume is in *xyz* space and *uv* represents the projections that are to be backprojected to the volume. Figure 1 shows the coordinate space. In the backprojection step, the volume *F* is reconstructed using the following equations [6]. From Equation 1, it is clear that each value in the 3D volume is independent and can be calculated in parallel.
1

where *W*_2_(*x*,*y*,*n*) represents the weight value and *u*(*x*,*y*,*n*) and *v*(*x*,*y*,*z*,*n*) represent the co-ordinates.
234

#### GPU computing

For many algorithms with massive parallelism, GPUs provided higher peak performance than CPUs. Initially GPUs were designed for processing graphics applications and games, but they have been increasingly used for scientific computing and biomedical applications such as Smith-Waterman alignment algorithm, protein folding, DNA sequencing, statistical phylogenetics, molecular dynamics, diffuse optical tomography and biological systems simulation [7–13].

GPUs have many parallel cores that run simultaneously and each core can run multiple threads. CT reconstruction has inherent features that can be parallelized. The sequential parts can be run on the CPU and the computationally intensive parallel parts can be accelerated on the GPU.

We have implemented FDK using two GPGPU languages: OpenCL and CUDA-C. While CUDA-C runs only on NVIDIA hardware, OpenCL is platform independent and runs on several hardware architectures including AMD, Intel, and NVIDIA. NVIDIA provides optimized libraries along with CUDA-C, which often results in better performance. Both CUDA-C and OpenCL support heterogeneous computing with separate host and device code. Both languages require minimal extensions to C/C++ programs. The accuracy of the results is of paramount importance in biomedical applications. We have shown that the results provided by GPU may have better precision over serial CPU code for floating point values [14].

#### Related work

There are several areas to explore to make the reconstruction faster. The first is to use a different algorithm. Authors have used this approach to obtain around 40 times speed up of reconstruction over traditional filtered backprojection [15, 16]. However, the quality of the reconstruction has been questioned [17]. Another area is to explore different parallel techniques and architectures. The intrinsic parallel nature of the algorithm makes it amenable to hardware acceleration for real-time processing. A popular hardware platform for parallel processing is to use GPUs. Attempts to use GPU hardware to accelerate CT algorithms date back to the early 90s when texture mapping hardware was used for 3D reconstruction [18]. Later Mueller and Xu used a GPU to accelerate backprojection by using accelerated graphics components [3, 19]. Zhao *et al.* [1] introduced an idea to allow larger datasets to fit in GPU memory. Noel *et al.* [20] used device memory to transfer all images and calculate intensity of a voxel. However accessing this memory can have long latency, so to avoid it, Knaup *et al.* [21] divided the total data into chunks to fit in shared memory. Mueller *et al.* [22] divided the processing by doing convolution on the CPU and backprojection on the GPU to reconstruct faster. The most similar work to ours is the Reconstruction Toolkit (RTK) [23], based on the Insight Toolkit [24]. Our approach is completely stand alone and does not require ITK or any other packages to operate. It makes use of the same inputs as those used by Fessler. Our CUDA-C code is compatible with nvcc, the NVIDIA C Compiler while that from RTK is not. Our OpenCL implementation is as optimized as the CUDA-C version and in fact produces superior results.

**Figure 2:**
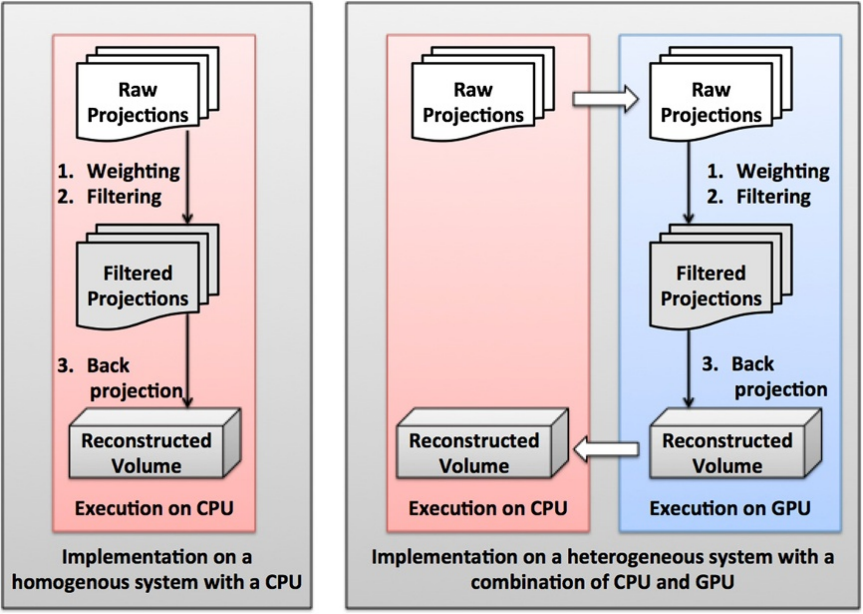
**Overview of serial CPU implementation and the implementation that makes use of a combination of CPU and GPU.**

Our approach can be seen as a combination of previous work. The implementation is divided into two parts with convolution on the CPU and backprojection on the GPU. In our implementation, we consider each pixel to be independent and load the full volume on the GPU. In contrast [25], considers all projections, but only part of the volume. We transfer the whole projection data to the GPU at an early stage and transfer the reconstructed volume back to the CPU at the end of all three steps: weighting, filtering and backprojection. The processing steps for the CPU and GPU implementations are shown in Figure 2. During backprojection, a large number of threads are launched on the GPU to compute each voxel in parallel. Each voxel is independently mapped to the final 3D volume.

This paper presents a more complete and consistent set of experiments and results than our previously published work [26]. All experiments in this paper are done on the same hardware for better comparison; the hardware is described in Section ‘Experimental results’. The OpenCL version is more advanced than in our previous publication and the best OpenCL implementation of backprojection available. The software described is now available for download [5].

### Implementations

We have several implementation of backprojection: 1) the MATLAB code originally writtend by Fessler *et al.* [4], 2) a version of Fessler’s code parallelized with MATLAB Parallel Computing Toolbox (PCT) 3) a serial implementation written in C, 4) the C implementation parallelized with OpenMP constructs [27], 5) a version that uses a combination of CPU and GPU written in CUDA-C that compiles with the NVIDIA compiler, nvcc, and 6) a version that uses a combination of CPU and GPU written in OpenCL.

We have implemented the FDK method in a basic processing chain in a pipelined fashion. The steps in the pipeline are: 1) load projection data, 2) ramp filter the weighted data and 3) backproject it to the final volume. Note that the structure of our code follows that of Fessler’s implementation. The input and output formats are also the same.

For the GPU implementations, different kernels are launched for different stages. Although the kernel calls are issued in a non-blocking manner, they are executed in series as each step needs to complete before the next can begin. In the filtering stage, different pixels for the same projection can be simultaneously filtered as there is no dependency between pixels. The filtering stage uses a Fast Fourier Transform (FFT). In the CUDA code we used the CUFFT available from NVIDIA [28] while for OpenCL we use Apple’s FFT package. The final step is to calculate voxel-based backprojection. Here each voxel is calculated in parallel by performing a matrix-vector product for each voxel in order to determine the corresponding projection value (see Equation 1). After all projections have been processed and mapped to the appropriate voxel, the final reconstructed volume is transferred to host memory. As memory transfer from host to device is expensive, transferring all the data to the GPU before the start of computation and transferring back the result after the final volume is reconstructed saves data transfer cycles. We use asynchronous data transfers to overlap data transfer with computation.

### Experimental results

We demonstrate the implementation of the FDK algorithm on two types of architectures: CPU and a combination of CPU and GPU. Details of the different hardware is summarized in Table 1. Note the difference in numbers of cores. As will be seen in the results section this is the largest contributor to performance since backprojection has a large number of independent compuations.

**Figure 3:**
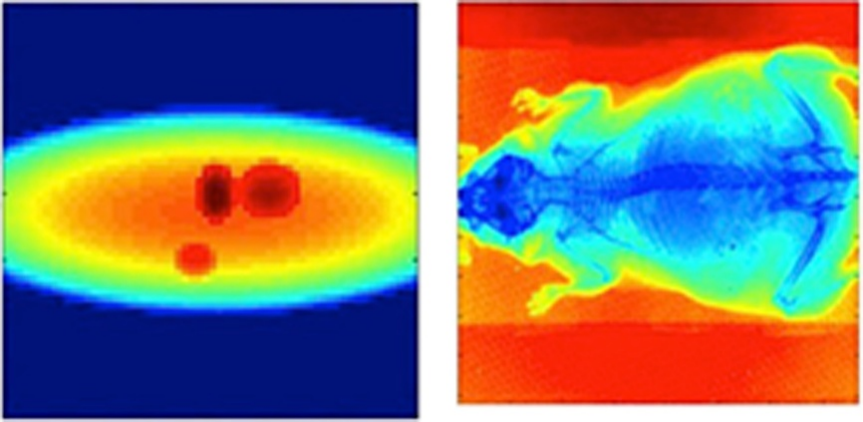
**A projection of the mathematical phantom (left) and the mouse phantom (right).**

We have implemented the FDK method. Relative performance is measured using two datasets. One, a synthetic mathematical phantom generated by MATLAB, has an input data size of 64 × 60 pixels with 72 projections to get a final volume of 64 × 60 × 50 voxels. The second is a mouse scan of 512 × 768 pixels with 361 projections. The dimensions of the output volume are 512 × 512 × 768. A single projection of the phantom and the animal scan is shown in Figure 3. This data was obtained and is presented with permission of Mass General Hospital.

**Table 1:** **Hardware details**

Processor	Clock	Number of	Cache	Memory
	speed	cores	size	size
Intel Xeon	2.00 GHz	6	15 MB	32 GB
E5-2620				
NVIDIA Tesla	1.15 GHz	448	768 KB	6 GB
C2075				
AMD Radeon	925 MHz	2048	768 KB	3 GB
HD 7970

The input and output sizes of the mathematical phantom are both 1MB. For the mouse scan, the sizes of the input and output projections are 542 MB and 768 MB respectively. Note that the code and therefore the run time only depend on the size of the data, not the content.

**Figure 4:**
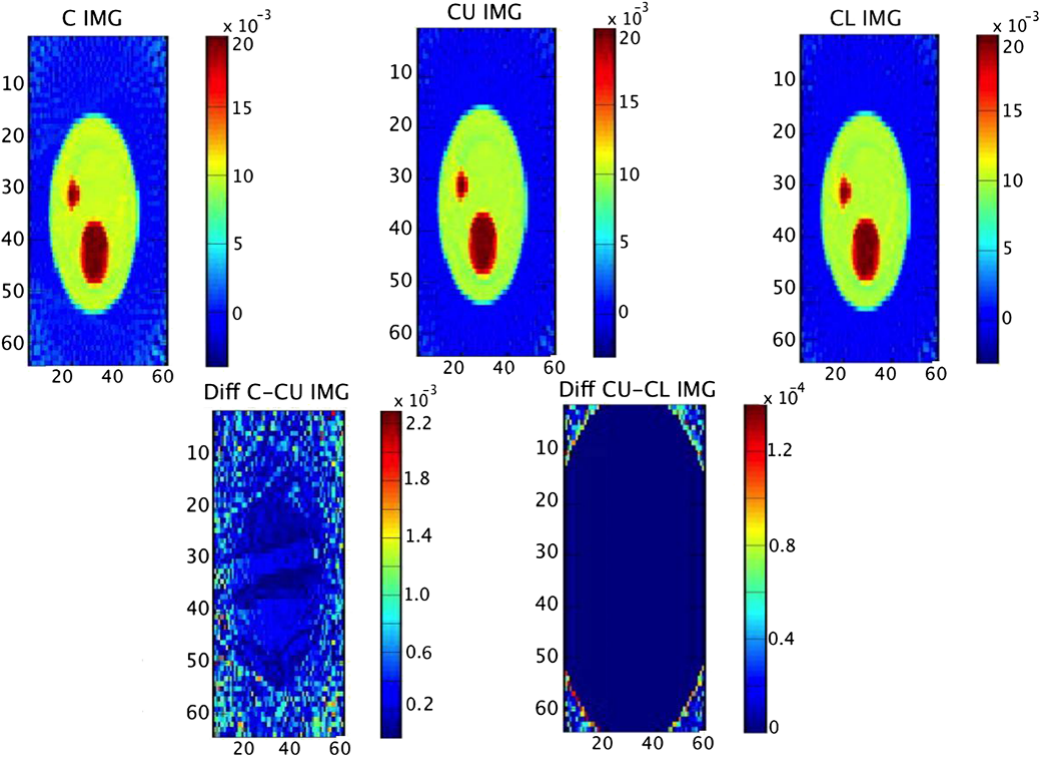
**Comparison of results obtained using C, OpenCL and CUDA-C run on phantom data.**

As mentioned earlier, the quality of reconstruction is important. To show that accuracy is not compromised, Figure 4 compares one slice of the final reconstructed volume in three implementations: 1) single threaded C, 2) OpenCL on NVIDIA and 3) CUDA-C on NVIDIA as well as the difference in values. Note that the difference in values is bounded by 2.2×10^-3^.

The performance of different implementations is listed in Table 2. Our performance data measure end-to-end execution time. For GPU implementations, they include data transfer times to and from the GPU as well as kernel execution times.

**Table 2:** **Performance of different implementations (in seconds)**

Dataset	Approach	Backprojection time	Total time	Speedup over MATLAB	Speedup over C
Phantom	MATLAB	51.06	51.11	–	–
Phantom	C	3.93	3.95	12.94	–
Phantom	C + OpenMP (4 threads)	0.85	0.89	57.43	4.44
Phantom	OpenCL (NVIDIA)	0.01	0.30	170.37	13.17
Phantom	CUDA (NVIDIA)	0.01	0.30	170.37	13.17
Phantom	OpenCL (AMD)	0.01	0.32	159.72	12.34
Mouse scan	MATLAB	33760.40	33777.33	–	–
Mouse scan	MATLAB PCT	22506.49	22513.90	1.5	–
Mouse scan	C	18451.77	18462.60	1.83	–
Mouse scan	C + OpenMP	5112.94	5615.65	6.01	3.29
Mouse scan	OpenCL (NVIDIA)	49.44	60.45	558.76	305.42
Mouse scan	CUDA (NVIDIA)	47.79	58.87	573.76	313.62
Mouse scan	OpenCL(AMD)	16.01	28.02	1205.47	658.91

**Figure 5:**
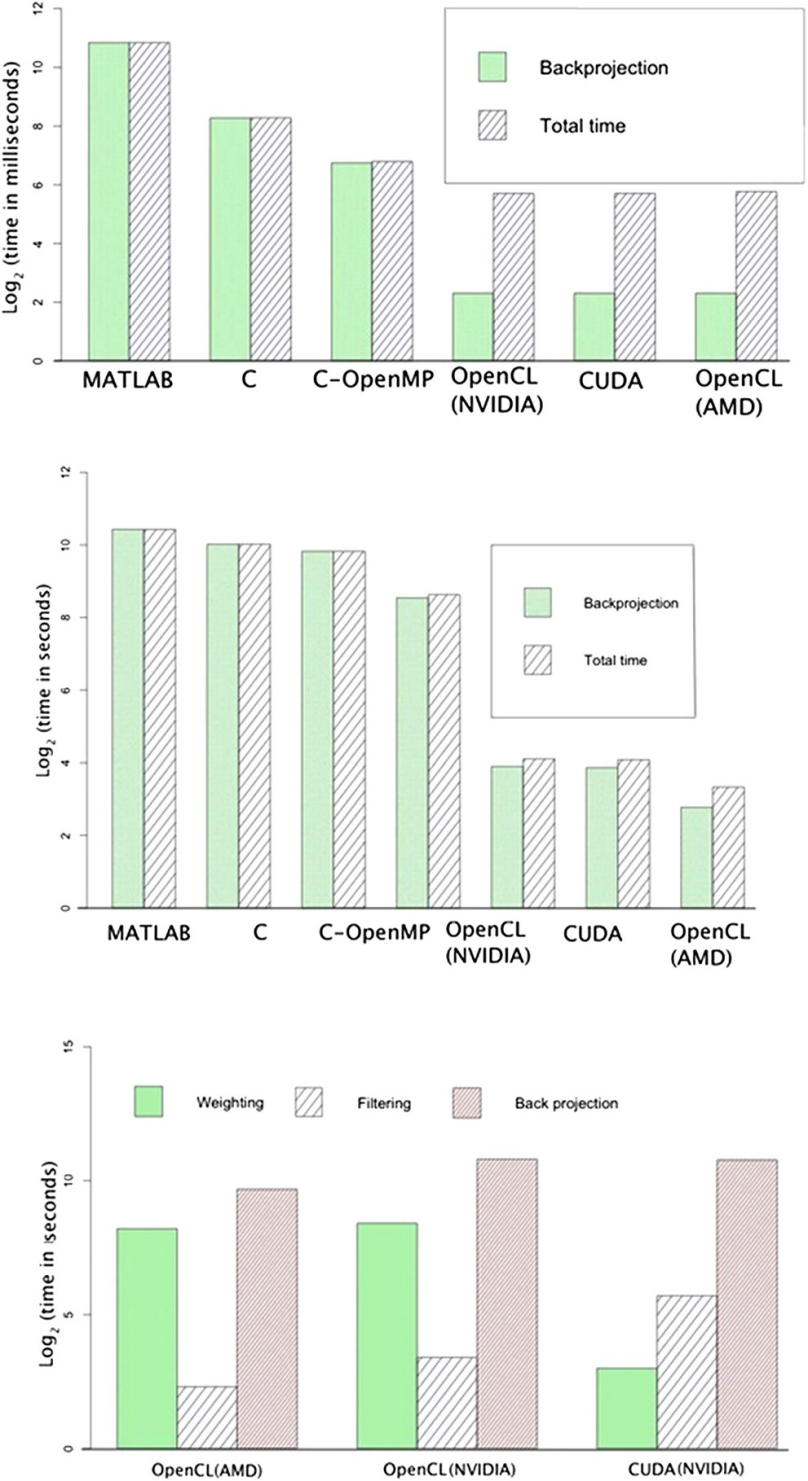
**Runtimes of different implementations applied to phantom data (top), mouse data (middle) and mouse data for each implementation component (bottom).**

It is evident that backprojection takes more than 99% of the total time in the serial MATLAB code. This is the motivation for parallelizing backprojection. The multi-threaded MATLAB implementation shows a speedup of 1.5x over serial MATLAB for the mouse scan data. The C implementation is 1.83 times faster than serial MATLAB, and the multithreaded C implementation with four threads is approximately 3.25 times faster again. Compared to the multithreaded C implementation, GPUs give the best performance. CUDA-C and OpenCL on the NVIDIA GPU we targeted both give a speedup of approximately 93 times over multi-threaded C. The fastest time of all was with OpenCL run on the AMD GPU. Here the speedup was 200 times compared to multithreaded C. Figure 5 top and middle show the runtime of different implementations on a logarithmic scale. The same OpenCL run on an NVIDIA GPU takes 60.45 seconds to reconstruct the image while it takes 28.02 seconds on the AMD GPU we used. Figure 5 bottom shows the runtime taken by each of the three stages of the algorithm on the two GPU cards: AMD and NVIDIA for two implementations: CUDA and OpenCL. Measured runtimes are given in Table 2.

## Conclusions

We have implemented the FDK algorithm [2], compatible with Fessler’s image reconstruction toolbox [4] and tested on two different architectures: CPU and a combination of CPU and GPU. Both NVIDIA and AMD GPUs have been used for performance evaluation. The performance of two GPU implementations in CUDA-C and OpenCL have been compared to MATLAB, Multithreaded MATLAB, and serial and multi-threaded C. The OpenCL implementation on the AMD card yields the largest speed up of 200x over multi-threaded C and three orders of magnitude over the original MATLAB code.

In the future, we will continue to improve our approach. After parallelizing backprojection, the new bottleneck is weighted filtering. We plan to investigate improved performance for the filtering stage. In addition, for the GPU implementations, only a subset of the number of launch configurations for kernels have been tested so far. The number of threads have been arbitrarily chosen. These issues will be investigated with auto-tuning. The data sizes that have been tested so far can be accommodated in the GPU memory, but for larger data sizes, streaming needs to be added to the current implementation. We plan to do so in future versions of the open source code.

## Availability and requirements

- Project name: Accelerating 3D CBCT with GPU
- Project home page: http://sourceforge.net/projects/acceleratecbct/
- Operating system(s): Linux
- Programming language: C with OpenMP, CUDA, OpenCL.
- Other requirements: CUDA compiler installed
- License: GPL
- Any restrictions to use by non-academics: Only those imposed already by the license.

## Availability of supporting data

All materials are available online. The source codes as well as input data phantom are released into the public domain. The documentation for the software pipeline is also included. This is available as Open Source software under the General Public License (GPL) version 2.0. as a part of the open source software [5].

## Abbreviations

CUDA: Compute unified device architecture
CT: Computed tomography
AMD: Advanced micro devices, Inc.
FDK: Feldkamp–Davis–Kress
GPU: Graphics processing unit
PCT: Parallel computing toolbox.
